# The discovery of the growth cone and its influence on the study of axon guidance

**DOI:** 10.3389/fnana.2015.00051

**Published:** 2015-05-15

**Authors:** Elisa Tamariz, Alfredo Varela-Echavarría

**Affiliations:** ^1^Instituto de Ciencias de la Salud, Universidad VeracruzanaXalapa, Mexico; ^2^Instituto de Neurobiología, Universidad Nacional Autónoma de MéxicoQuerétaro, Mexico

**Keywords:** chemotropic, neurotropic, brain, development

## Abstract

For over a century, there has been a great deal of interest in understanding how neural connectivity is established during development and regeneration. Interest in the latter arises from the possibility that knowledge of this process can be used to re-establish lost connections after lesion or neurodegeneration. At the end of the XIX century, Santiago Ramón y Cajal discovered that the distal tip of growing axons contained a structure that he called the growth cone. He proposed that this structure enabled the axon’s oriented growth in response to attractants, now known as chemotropic molecules. He further proposed that the physical properties of the surrounding tissues could influence the growth cone and the direction of growth. This seminal discovery afforded a plausible explanation for directed axonal growth and has led to the discovery of axon guidance mechanisms that include diffusible attractants and repellants and guidance cues anchored to cell membranes or extracellular matrix. In this review the major events in the development of this field are discussed.

## An Influential and Unsuspected Discovery

In the study of nature, great discoveries are often made in the context of heated debates regarding alternate explanations for biological phenomena. On other occasions, discoveries reveal hidden or previously unnoticed aspects of the systems under study. The latter is the case for the discovery of the growth cone, a structure that would strongly influence neuroscientific work, particularly neurodevelopmental research. This structure, whose existence was unsuspected, is located at the distal tips of growing axons. It was discovered by Santiago Ramón y Cajal in 1890 when he delved into the study of chick embryos in his search for evidence supporting the neuron doctrine, then at the center of a raging controversy among the most prominent neuroscientists. This discovery attests to the keen analytical eye of Cajal and shortly afterwards he put it into the context of the “neurotropic” hypothesis that played an instrumental role in settling the rival “neuronist” and “reticularist” theories for the organization of the nervous system (Guillery, 2007). The discovery of the growth cone prompted new lines of research, but the hypothesis exerted its most profound influence on neurodevelopmental research only about a hundred years later.

In this essay, we will review the major developments in the field of axon guidance into which the concept of the growth cone was inserted, and we will discuss its influence on the field. Various and very through historical accounts of the discovery of the growth cone have been written in recent years (Sotelo, 2002; de Castro et al., 2007; Garcia-Marin et al., 2009); here the focus will be on the development and evolution of the concept of growth cone and its influence on neuroscientific thought and knowledge. The chronology of the major events, mainly related to axon guidance during embryonic development, will be maintained throughout this brief essay; parallel developments, however, will sometimes be described in consecutive sections.

## Neuroscientific Research at the Time of the Discovery of the Growth Cone

The idea that axons were part of nerve cells and that these cells were the individual morphological and functional units that composed the nervous system, was put forward by His and Forel in 1887 (Cajal, 1907; Partsalis et al., 2013). His derived his ideas from developmental studies in which he observed the continuity from post-mitotic neurons to growing axons (Sotelo, 2002). Forel, in turn, observed atrophy of cranial motor neurons after severing their nerve roots (Sotelo, 2002). This notion contrasted with the prevalent view at the time that the nervous system consisted of a group of nerve cells connected in a “continuous network” which, in turn, derived from previous discoveries made in the context of the “cell theory”, in which muscle cells were recognized as multinucleated cellular entities (Guillery, 2007).

The work of His, Forel, Gower, Kollicker, Retzius, Gehutchen, von Lenhossek, Nansen and Cajal himself contributed to the neuron doctrine which was formally enunciated by Waldeyer-Hartz in 1891 (Guillery, 2007). It viewed the neuron as the structural, ontogenetic, functional and trophic unit of nervous tissue and further stated that individual neurons communicated via contiguous interactions and not by fusion between them. This idea was later complemented by Cajal and Van Gehutchen’s notion of dynamic polarization which recognized dendrites and cell bodies as the receptive portion of nerve cells and the axon as their effector component. In contrast, in the context of the reticularistic theory, the nervous system was viewed as a continuous system in which all nerve cells anastomosed into a single network (Guillery, 2007).

These opposing views of the structure and workings of the nervous system, confronted each other at both the morphological and functional levels, but they also needed adequate explanations at the ontogenetic level. That is, accurate descriptions of the structure of the nervous system required consistent mechanisms of the way in which it is formed during embryonic development. While the reticular theory invoked fusion of individual cellular units to generate a fully anatomosed nervous system (Hensen’s “catenary” or “polygenic” theory) (Cajal, 1929a; Partsalis et al., 2013), the neuronist theory proposed that axons grew out of the neuronal cell bodies and reached other neurons but never fused with them. It was in this atmosphere that the discovery of the growth cone was made.

## Enter the Growth Cone

**Figure 1:**
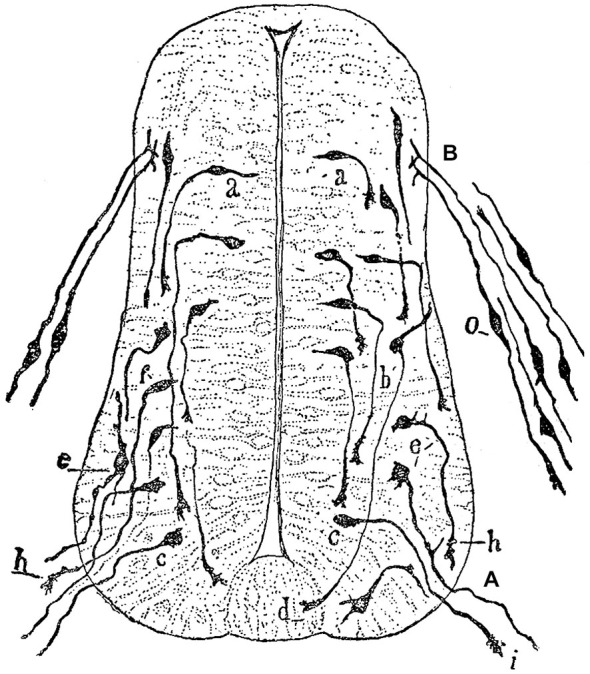
**Drawing by Cajal of a spinal cord section of a three-day old chick embryo. (A)** Ventral nerve root; **(B)** Posterior nerve root; **(a)**, young nerve cells; **(b)**, more developed cells that probably correspond to commissural neurons; **(c)** piriform cell of the ventral nerve root; **(d)** growth cone of a commissural axon; **(e)** radicular cell with rudiments of protoplasmic branches; **(h,i)** growth cones of ventral nerve root axons; **(o)** dorsal root ganglion cells. Figure taken and legend translated from Cajal (1929b).

**Figure 2:**
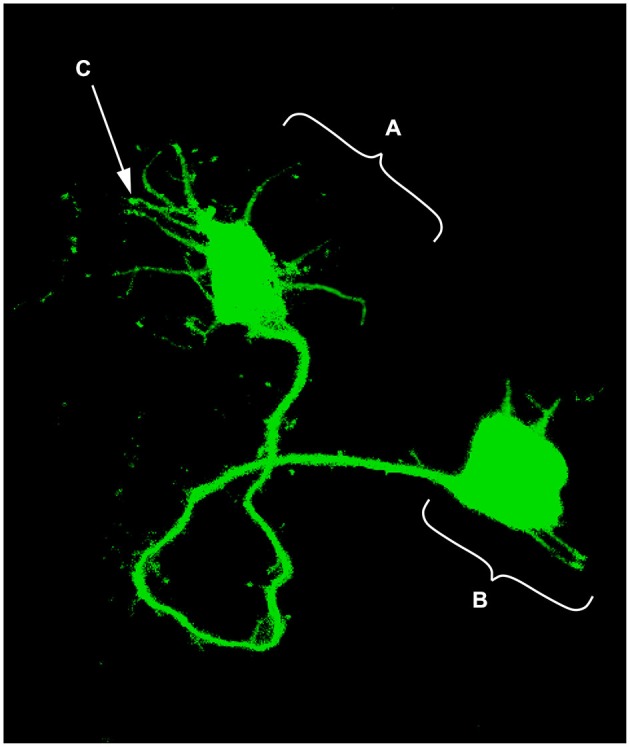
**Single neuron electroporated with a green fluorescent protein (GFP) vector in an embryonic chick hindbrain. (A)** Growth cone; **(B)** Neuronal soma; **(C)** Filopodia (digitally enhanced for better visualization).

**Figure 3:**
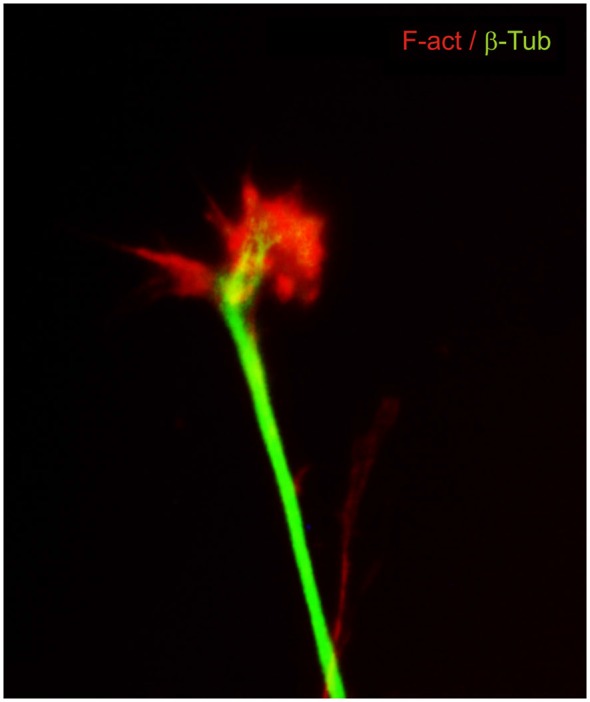
**Dorsal root ganglion neuron in culture immunostained for cytoskeletal components**. β-III tubulin microtubules (green) are located mainly in the axon shaft and in the central part of the growth cone. F-actin filaments (red) are located at the leading edge and in the lamellipodia and filopodia.

The idea that axons grew out of nerve cells was generally accepted at that time and stemmed primarily from the influential work of His. However, there was neither a paradigm of directional growth nor any idea that the end of the growing axons could respond to any feature of the tissue it traversed. Cajal turned to the study of chick embryo preparations seeking evidence in support of what later would be called the “neuronistic” view of the organization of nerve tissue, as stated above. This was only possible by using the silver staining method of Golgi with improvements by Cajal (for a detailed account of the development of these methods see (Garcia-Marin et al., 2009)). Since it was found that myelination limited the reach of the Golgi method, Cajal worked with younger and younger embryos. This led him to study spinal cord preparations of three- and four-day old chick embryos in which he described the stereotypical behavior of growing axons (Figure 1). He soon observed that the distal tip of the axons of commissural neurons widened into a “cone-like lump with a peripheral base” decorated by triangular or short thorny processes (Cajal, 1890). This description refers to what is currently considered the main morphological feature of a growth cone, the widened end of a growing axon with lamellipodia and filopodia at its leading edge (Figure 2). Using complementary information obtained by two histological methods, the Golgi method and the reduced silver nitrate method, Cajal was able to identify with more detail the structure of the growth cone, thus describing it as containing a “neurofibrillar bundle”, which is now known to be composed of actin filaments (AFs) and microtubules (Figure 3; Garcia-Marin et al., 2009).

It was thus that Cajal, walking the untrodden path of scientific inquiry in the central nervous system, identified a structure which he referred to using various names, eventually settling on the term “growth cone”.

## The Chemotactic or Neurotropic Hypothesis

Concomitant with the discovery of the growth cone, Cajal observed that the behavior of growing nerve fibers was reproducible but depended on their origin: commissural axons navigated ventrally from the dorsal aspect of the spinal cord and across to its contralateral side, axons of motor neurons exited ventrally, and fibers of neurons from the dorsal root ganglia entered into the dorsal spinal region (Figure 1). These observations led Cajal to consider that nerve fibers “adopt predetermined directions and establish connections with defined neural or extra neural elements… without deviations or errors, as if guided by an intelligent force” (Cajal, 1890).

On occasion of a cholera outbreak in Valencia in 1885 where he was then Professor at the University of Valencia, Cajal had a brief but fruitful excursion into microbiology which acquainted him with contemporary knowledge regarding the oriented movement of leucocytes toward bacteria guided by gradients of substances produced by the latter (Sotelo, 2002). This is thought to have strongly influenced and inspired the 1890 ideas of directed axonal growth in response to chemical signals that diffused from their targets that he would later include in formulating the neurotropic hypothesis. In the early stages of the formulation of his postulates regarding axon growth, he envisioned diffusible attractive and repulsive signals that impinged upon growth cones thus determining their direction of growth. In later and more mature descriptions, the repellants were not part of the hypothesis. Moreover, based on the observation of the morphological diversity of growth cones in different regions of the spinal cord, Cajal proposed that the growth cone was motile and responsive to the nature of the substrate, which also determined the route axons would take as they extended (Cajal, 1899; Garcia-Marin et al., 2009). Both components of his postulate, the response to graded, diffusible chemical signals and the interaction with the substrate, would, in time, prove to be very accurate.

At a scientific meeting in the same year that Cajal first described the growth cone, and also based on observations in embryonic chick tissue, Michael von Lenhossek proposed that the “free-end” of growing nerve cell fibers were endowed by a “miraculous energy” that allowed them to extend along their pathway (de Castro et al., 2007). Although no mention was made of a specialized structure at the fiber end, the general interpretation of its relevance in the directed growth of nerve cell fibers coincided with that of Cajal.

Cajal’s remarkable ability to extract mechanistic explanations of the directed growth of axons based on snapshots of developmental tissue preparations may be difficult to appreciate today. It is a great achievement of his acute observation ability. This hypothesis led Cajal and others to study the neurotropic mechanisms that were paramount in his postulate (de Castro et al., 2007). As embryonic tissue did not allow him to obtain the proof he wanted, he turned instead to the study of regenerating peripheral axons. In this work, Cajal succeeded in demonstrating that regenerating axons grew towards grafted nerves only when living Schwann cells were present and not when these nerves had been treated with chloroform, thus killing the cells (Sotelo, 2002). Although these findings provided support for the neurotropic hypothesis, the definitive proof would come only about one hundred years later.

## Immediate Effects of the Neurotropic Hypothesis

The neurotropic hypothesis, with the growth cone at its vanguard, significantly strengthened Cajal’s argument for the validity of the neuron theory. It not only provided a mechanistic view of how nerve connections are established between individual cellular units, but it also consolidated the view of the nerve cell, the neuron, as first named by Waldeyer, as the basic structural and functional unit of the nervous tissue. Although definitive proof of the neuron theory was only obtained half a century later by Estable and DeRobertis (Guillery, 2007), it was a conceptual leap that provided the neuron as a useful reductionist tool that persists in most areas of present day neuroscientific research. The neuron view, in a subsequent turn of the wheel, fueled developmental studies that addressed the behavior of growing fibers from individual nerve cells, which were by then considered to remain independent even as they established contacts with other neurons or muscles through structures which would later be named “synaptic contacts” by Sherrington (1906).

## Study of the Growth Cone without the Knowledge of Chemotropic Effectors

After the original proposal, the neurotropic hypothesis languished as no evidence was produced which fully confirmed the notion of the “diffusible signals” envisioned by Cajal. The growth cone, however, continued to be a useful concept intimately associated with the observed behavior of growing axons. It was thus during this apparent impasse, that different neuroscientists initiated various lines of research into aspects of the growth cone, such as its behavior, morphology, ultrastructure, dynamics, molecular composition and interaction with the substrate.

Observation of neuron fiber outgrowth was not possible until the first decade of the twentieth century when Ross Harrison developed a culture method for neural tissue explants (Harrison, 1910). He observed that the elongation of processes from the leading edge of the growth cone is involved in the motility of the structure and described two phenomena in nerve development, “(a) the formation of the primitive nerve fiber through extension of the neuroblastic protoplasm into a filament—protoplasmic movement; (b) the formation of the neurofibrillae within the filament—tissue differentiation…” (Harrison, 1910; Keshishian, 2004). This evidence finally confirmed that axons elongate from a single cell body, further supporting the neuron doctrine of Cajal and setting aside the syncytial theory of Victor Hensen and that of the Schwann cell origin of the axons held by Theodor Schwann (Garcia-Marin et al., 2009). This secured the growth cone as the leading actor in axon outgrowth.

Since the methods used in the early studies with cultured nervous tissue only allowed the growth of axons on solid substrates while submerged in liquid medium, research on axon guidance went mostly into the analysis of the interaction of axons and growth cones with the substrate relegating the chemotropic hypothesis to the background for decades. This led Weiss in 1941 (Weiss, 1941) to propose the “contact guidance” hypothesis, which postulated the need for a contact surface with specific matching between the axons and their substrate situated in the intercellular matrix immediately adjoining cells but not at a distance. Several reports thereafter focused on describing the motility of the growth cones. Phase-contrast microscopy and time-lapse imaging improved the observation of neuron outgrowth (Pomerat, 1951), and *in vivo* observations suggested similarities of growth cone displacement with that of pseudopodia of amoeba and macrophages (Pomerat, 1951; Hughes, 1953; Nakai, 1956). Electron microscopy of projections from dorsal root ganglia entering the neural tube of rabbit embryos, allowed the filamentous contents of axons and growth cones to be observed, revealing large spindle-shaped varicosities containing smooth reticulum, mitochondria, dense bodies, neurofilaments and microfilaments (Tennyson, 1970). This description supported Cajal’s findings, in Golgi silver-impregnated dorsal root ganglion, of neurons entering the spinal cord (Cajal, 1909).

Moreover, electron microscopy revealed the first detailed distribution of cytoskeletal components within the growth cone, with neurofilaments in its central area and in the microspikes or filopodia; microtubules were mainly found in the axon with a few protruding into the growth cone (Yamada et al., 1970). A fine filamentous network was also described in the lamelipodia and filopodia and occasionally thin microtubule (MT) filaments were observed invading the filopodia (Yamada et al., 1970; Yamada and Wessells, 1971). In the same reports, and using inhibitors of polymerization of cytoskeletal components such as cytochalasin B and colchicine, Yamada and co-workers showed that inhibition of AF polymerization induced retraction of growth cones while high concentrations of colchicine inhibited MT depolymerization and eventually induced axon retraction. This demonstrated for the first time the relevance of AF and MT polymerization in growth cone formation and neurite outgrowth (Yamada et al., 1970; Yamada and Wessells, 1971). These results led to the conclusion that AF are important for growth cone shape and that MT are essential for axon structure, thus opening the field to a molecular explanation of growth cone motility.

## The Second Age of the Neurotropic Hypothesis and the Identification of Chemotropic Molecules

In parallel to the advances on the cytoskeletal dynamics of the growth cone, the neurotropic hypothesis has recently experienced a revival. The first major development that marked this revival was the finding by Andrew Lumsden and Alun Davies of a signal that elicited and attracted the growth of neurites from trigeminal sensory axons (Lumsden and Davies, 1986). In keeping with one of the original postulates of Cajal, the final target, in this case the whisker pad epithelium, exerted the described effect on the sensory neurons that innervate this tissue. Soon thereafter, technological advances including the great analytical power of biochemistry and molecular biology and the ability to manipulate embryonic tissue *in vitro* and *in vivo*, allowed the first truly chemotropic molecules for growing axons to be discovered. Using the same culture method devised by Lumsden and Davies, Tessier-Lavigne and collaborators detected, in the floor plate of chick embryos, diffusible signals that attracted commissural axons (Tessier-Lavigne et al., 1988). The attractive signal was identified a few years later as a member of a family of proteins named netrins by the same group (Kennedy et al., 1994; Serafini et al., 1994). Since then, more protein families have been identified that have chemotropic effects on many different axon types in invertebrates and vertebrates. These include semaphorins (the first family known to include chemorepellants), slits, and some proteins that were previously known to have other biological effects, such as Shh, FGF8, and HGF (Tessier-Lavigne and Goodman, 1996; Varela-Echavarría and Guthrie, 1997; Huber et al., 2003). The far-reaching findings that have been made from the study of these axon guidance molecules include: that among them there are both attractants and repellants, that the same molecule may have both effects on different axon types, that the same axon type responds specifically to different guidance cues and that its ability to respond to these cues changes during development. Moreover, for each family of guidance cues, the receptor complexes that allow the growth cone to detect the signals and the main components of the intracellular cascades involved in the axon response have been identified (Huber et al., 2003; Bashaw and Klein, 2010).

## Interaction of the Growth Cone with Signals Anchored to the Substrate

Numerous studies have also revealed that responses to chemotropic guidance cues necessarily involve interaction of the growth cone with the substrate, thus confirming one of Cajal’s ideas regarding nerve cell fiber growth. Families such as cell adhesion proteins of the immunoglobulin superfamily (IgCAMs), integrins, and extracellular matrix proteins such as fibronectins, laminins and thrombospondins mediate the anchoring of axons and growth cones to the substrate during their growth and elicit cytoskeletal responses (Neugebauer et al., 1991; Osterhout et al., 1992; Myers et al., 2011). For example, during growth cone movements, weak interactions with the substrate result in a slow, forward movement of cytoskeletal components, high retrograde flow, and no tension at the adhesion sites (Suter and Forscher, 2000). In contrast, strong adhesion mediated by NCAM, N-cadherin and integrins, translates the myosin-driven AF flow into forward growth cone movement which, in turn, attenuates retrograde flow and enhances the actomyosin-mediated traction force pulling the growth cone forward (Suter and Forscher, 2000; Suter and Miller, 2011). In this sense, the adhesion of the growth cone to the substrate also mediates the responses to guidance cues; repulsive effects of the neurotropic proteins Slit via their receptors Robo, inhibit N-cadherin-mediated cell adhesion (Rhee et al., 2002). On the other hand, several lines of evidence have shown that DCC, a netrin-1 receptor, interacts directly with kinases involved in regulating adhesion, such as FAK, Src and Fyn and that their disruption blocks axon attraction and turning in response to netrin1 (Liu et al., 2004; Ren et al., 2004). Other evidence shows a relationship between the adhesion proteins integrins and L1 with Plexins and Neuropilins, which are components of the receptor complexes of semaphorins (Castellani et al., 2002; Barberis et al., 2004; Valdembri et al., 2009; Seerapu et al., 2013).

A particular type of interaction between the growth cone and its target cells was envisioned by Sperry in 1963 as part of the “chemoaffinity hypothesis” that had been proposed earlier and had matured for over two decades (Sperry, 1963). He proposed that axons and neurons carry chemical tags that endow them with specific affinities for other neurons thus mediating selective “attachment” to them. Furthermore, the hypothesis also included gradients of chemical signals that explained the topographic projection of retinal axons into the tectum. A molecular explanation of the evidence supporting this hypothesis came with the discovery of ephrins and their receptors, both of which are membrane- anchored proteins that fit the definition for the chemical graded tags of Sperry’s postulates (Cheng et al., 1995; Drescher et al., 1995).

## From the Molecular Basis of Growth Cone Motility to Mechanistic Explanations of its Response to Guidance Cues

Several decades of research on the growth cone cytoskeleton have revealed the relevance of the dynamic interplay between AF and MT during axon growth. Immunofluorescent techniques and optical resolution methods have confirmed the differential distribution of cytoskeletal components at the growth cone and have provided exquisite details of the structure and function of the cytoskeleton during dynamic behavior (Gomez and Letourneau, 2014).

Based on these studies, three main regions with differential distribution of AF and MT are currently recognized in the growth cone and can be visualized by Differential Interference Contrast (DIC) microscopy. The peripheral region (P), composed of filopodia and lamellipodia, is characterized as an AF-rich region with filaments assembling at the filopodial tips and a meshwork-like array of AF in the lamellipodia. The transitional domain (T) contains arc-like AF structures delimiting the central domain (C) and some advancing MT overlapping with AF. The C region is rich in MT with their polymerization-plus ends facing the T zone and with punctate F-actin distribution (Schaefer et al., 2002; Dent and Gertler, 2003; Suter and Miller, 2011). Abundant evidence has shown that interactions between AF and MT in the growth cones are essential for axon outgrowth, growth cone turning, and response to guidance cues for pathfinding and branching (Letourneau et al., 1987; Challacombe et al., 1996; Dent and Kalil, 2001; Buck and Zheng, 2002; Schaefer et al., 2002, 2008; Lee and Suter, 2008; Geraldo and Gordon-Weeks, 2009; Suter and Miller, 2011).

The studies on the dynamics of the growth cone cytoskeleton have also contributed to our understanding of the molecular mechanisms underlying the response to guidance cues. Early reports using Ti1 neurons of limb buds from embryonic grasshopper first described the role of actin polymerization for oriented projection of the growth cone (Bentley and Toroian-Raymond, 1986). One year later, Letourneau and co-workers published the seminal concept of the two forces exerted at the growth cone, the “push” of MT and the “pull” of AF, which has been instrumental in understanding growth cone motility and response to guidance cues (Letourneau et al., 1987). At present, it is known that the rate of F-actin polymerization and retrograde actin flow toward the base of the filopodia affects the rate of extension of filopodia and lamellipodia and therefore the translocation of the growth cone (Forscher and Smith, 1988; Lin and Forscher, 1995; Mallavarapu and Mitchison, 1999). ATP-actin monomers are assembled into filaments near the membrane in the distal P region of growth cones, and in a myosin motor-driven process, are transported rearward into the T region, where filaments are severed and ADP-actin monomers are recycled into new polymerizing filaments (Lin and Forscher, 1995; Dent and Gertler, 2003). The rearward transport is linked to interactions with the substrate via cell adhesion and extracellular matrix proteins (Dent and Gertler, 2003; Suter and Miller, 2011). When growth cones turn in response to positive guidance cues, MT selectively invade the axon branches that redirect toward the guidance signal, and a local rearrangement and advance of MT is observed in the filopodia that become stabilized towards the direction of turn (Sabry et al., 1991; Bentley and O’Connor, 1994; Lin and Forscher, 1995). The positive guidance cues polarize the formation of filopodia in the growth cone and stabilize their extensions by decreasing retrograde F-actin flow and depolymerization of AF. Conversely, negative guidance cues inhibit the protrusive activity of AF and MT polymerization at the growth cone (Gallo and Letourneau, 2004).

## Unifying Views of Growth Cone Function and its Interaction with the Environment

**Figure 4:**
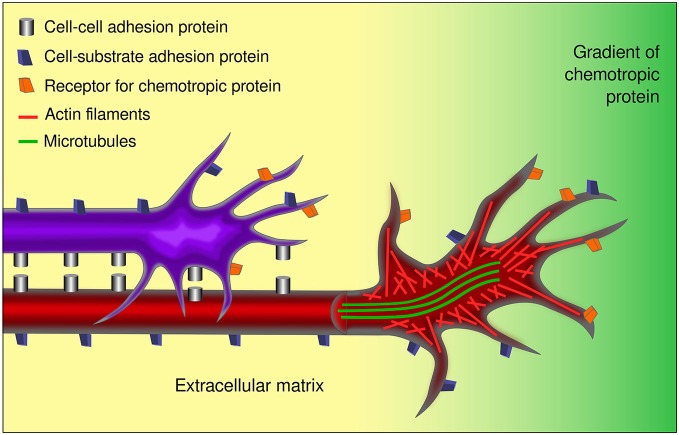
**Aspects involved in the response of growth cones to guidance cues**. Growth cones respond to several external factors including chemotropic proteins and signals anchored to cell or axon membranes and to the extracellular matrix. Growth cones integrate the information conveyed by the receptors to the various guidance signals inducing the changes in the cytoskeleton associated to axon navigation. The cell-cell and cell-substrate interactions involved, make axon pathfinding a complex and multifactorial event. In the figure, a pioneer axon is shown (red) that interacts with its substrate (yellow) and responds to a chemotropic signal (green gradient). A follower axon (purple) interacts additionally with the pioneer axon. The differential distribution of microtubules and actin are indicated in the open view of the red axon.

The concept of growth cone, together with diverse technological and methodological developments in the field, has led to the identification of chemotropic molecules, components of the extracellular matrix, and integral membrane proteins that determine axon pathfinding. In a nutshell, diffusible guidance cues may act as attractants or repellants for axons by impinging upon growth cones, while signals anchored to the substrate may permit, inhibit, or enhance axon growth. Together, the response to these signals, mediated by membrane receptors on the growth cone, is accompanied by changes in cytoskeletal organization of the growth cone and its adhesion to the substrate as it courses through the developing brain (Figure 4). Current knowledge confirms that the instructive directional signals of both neurotropic proteins and contact guidance cues are integrated by growth cones, resulting in stereotypical axon route finding (Myers and Gomez, 2011; Bonanomi et al., 2012; Moore et al., 2012; Leung et al., 2013; Garcia-Peña et al., 2014).

Although this picture closely mirrors Cajal’s chemotropic hypothesis in several respects, significant diferences between them exist; namely, Cajal proposed that the final targets were the source of attractive signals for growing axons and that “negative neurotropic substances” did not appear to participate in their growth, changing his initial view on the matter (de Castro et al., 2007). While final targets have been shown to exert attractive effects, intermediate targets also secrete chemotropic molecules with essential roles. Moreover, as stated above, chemotropic proteins include repellants as well as attractants.

## Future Challenges

**Figure 5:**
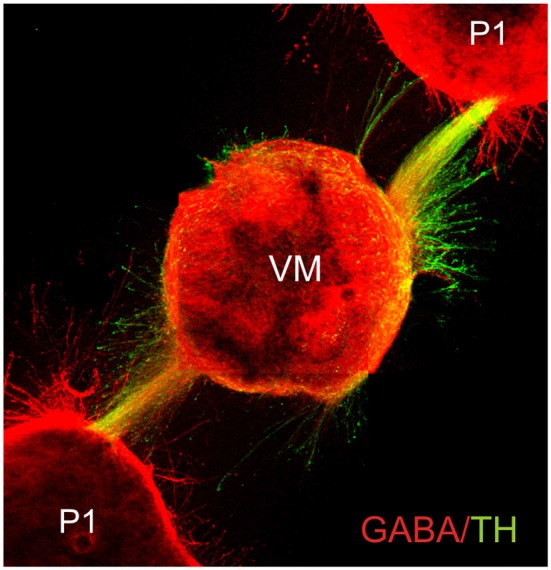
**Fasciculated projection of GABA^+^ and dopaminergic (TH^+^) axons in culture**. E13.5 rat embryo pretectum (P1) explants exert an attractive effect upon axon GABA/TH fascicles growing from ventral midbrain explants (VM) in collagen gel cultures. Cultured gels were immunostained for GABA (red) and TH (green) (Garcia-Peña et al., 2014).

Complex axon guidance mechanisms involving several, simultaneous signals have been documented. Many challenges lie ahead, however, as the efforts to understand the control of axon pathfinding have been directed only to a handful of axon types in the developing nervous system. Formidable problems are posed by neuronal types such as noradrenergic neurons of the locus coeruleus whose fibers project into practically all major brain areas. Although the same general principles of axon guidance are likely to operate in the projection to each of these areas, understanding their coordinated projection will require a considerable effort. Moreover, the complexity and dynamics of the substrates traversed by growing axons has made this a more difficult issue to study. In this regard, important facts are that most axons appear to interact with other axons during their route finding (Figures 4, 5), and that the response to chemotropic molecules is modulated by strong interactions with the substrate. For example, we have observed interaction between axons from opposite sides of the developing mouse hindbrain that is relevant for their decussation (Sandoval-Minero and Varela-Echavarría, 2008) and the anatomical coupling of sensory projections to motor axons influences sensory axon projection (Wang et al., 2011). Moreover, our group has recently shown that ascending midbrain dopaminergic axons grow in close apposition to descending GAD65-positive axons and that this interaction appears to participate in stereotypical dopaminergic projection (Garcia-Peña et al., 2014). We also observed that previously known misprojections of dopaminergic axons in embryos lacking the chemotropic molecules Slit1 and Slit2 or their receptors Robo1 and Robo2 (Dugan et al., 2011) were accompanied by severe alterations of the underlying GAD65 axon scaffold. From our results, it follows that the dopaminergic guidance defects in these embryos may be secondary to the alteration in the pre-existing GAD65 scaffold and that many such interactions may be taking place between other axon types throughout the developing brain. Hence, the conclusions of previous studies addressing the role of chemotropic guidance cues in the projection of dopaminergic and other axon types may require re-evaluation to incorporate the effect of the contact-mediated interaction with other axons or cells along their pathway.

On another matter, recent evidence suggests that signals that guide axons can be not only chemical but also physical properties of the substrate such as its nanotopography and stiffness, re-opening the field to what Weiss defined as “selective conduction” (Weiss, 1947). Modern micro- and nanofabrication techniques are contributing to an understanding of how physical cues are involved, since earlier experiments with aligned extracellular components or cells did not allow detection of chemical and physical influences on growth cones and axon growth (Ebendal, 1976; Hynes et al., 1986; Alexander et al., 2006). Substrate stiffness is also an element that impinges on growth cone behavior, as substrate rigidity can affect axon branching and extension (Flanagan et al., 2002; Kostic et al., 2007; Leach et al., 2007). Interestingly, the response to stiffness varies between neuronal types; while substrate stiffness modulates axon growth of DRG neurons, hippocampal neurons seem to be insensitive to it (Koch et al., 2012). How neurons sense rigidity, however, remains unknown and is under intense study (Hoffman-Kim et al., 2010; Moore and Sheetz, 2011; Suter and Miller, 2011; Dupin et al., 2013).

## Concluding Remarks

The long history of the growth cone concept has led us to discover many mechanisms involved in axon pathfinding during development. Additional evidence reveals that regenerating axons respond to signals in their environment following the same general principles. This has prompted efforts to use the information derived from developmental studies to elicit and guide the growth of axons in adult models of human diseases in neuro-regenerative medicine (Díaz-Martínez et al., 2013). This field is poised for important developments with many potential applications and a significant impact on human health.

The discovery of the growth cone and the formulation of the chemotropic hypothesis constitute two of the towering achievements of Cajal. Despite their shortcomings owing to the limited knowledge available at the time they were proposed, the concept of the growth cone and the chemotropic hypothesis, provided a framework to a complex type of processes in a way that took over a hundred years to fully understand.

## Conflict of Interest Statement

The authors declare that the research was conducted in the absence of any commercial or financial relationships that could be construed as a potential conflict of interest.
